# Pediatric urology: An emerging subspeciality

**DOI:** 10.4103/0970-1591.36893

**Published:** 2007

**Authors:** Shyam Joshi

**Affiliations:** Jaslok Hospital, Mumbai, India

I have enjoyed guest editing this special symposium on Pediatric Urology. Pediatric urology has always been very close to my heart. It is one of my dreams to see this speciality become established superspeciality in India like in United States of America. With the establishment of American Academy of Pediatrics – Urology section (AAP Uro) and European Society of Pediatric Urology this speciality has firmly established its role as superspeciality in the developed world. ESPU has an exit examination at the end two years of specialized training and the AAP is planning a similar exit exam in future. Situation in the developing world and in India continues to be significantly different. The pediatric urological diseases are managed both by the pediatric surgeons and the urologists. This turf war continues because pediatric surgeons usually treat children below age of 12 years in all government and teaching medical colleges in India. This has had an adverse effect on the growth of pediatric urology as subspeciality. Pediatric surgeons have given up long ago cardiac and neurosurgery because of extensive technical and scientific development in this field. Urology today is no exception. Development of endourology, urodynamics and renal transplantation work requires specialized training and skills which cannot be learnt during a pediatric general surgical training. In this issue, Dr. Oak, Professor of pediatric surgery from Mumbai and Prof. Paddy Dewan, a pediatric urologist from Melbourne, Australia have deliberated on the issue of "Training of pediatric urologist". It is obvious that it would require a multidisciplinary approach requiring support of Pediatricians, Endocrinologists, Nephrologists, Pediatric Anaesthetists and supporting nursing and paramedical staff. A pediatric surgeon who intends to pursue his career as pediatric urologist would need an additional training in the field of Endourology, urodynamics and renal transplantation. While, an urologist would need training regarding basic principles of pediatric general surgery. Instead of fighting a turf war, it would be better if the respective surgical societies of pediatrics and urology can come together and device a syllabus for a structured training programme. These societies would then need to identify units / institutions which can undertake such training.

This issue concentrates on some of the important advances which have been made in the field of pediatric urology. The treatment of VUR is better standardized today and the role of surgery is clearly defined. Drs. Dave and Khoury from Torronto Hospital for Sick Children provide evidence based approach for management of VUR. Dr. Atul Thakre elaborates the evolving role of laparoscopy in antireflux surgery. Dr. Pramod Reddy from Cincinnati have discussed very lucidly the advances in the field of Uroradiology.

Urolithiasis in children is not an uncommon problem in our part of the world and Prof. Rizwi etal from SIUT Karachi and Dr. Mahesh Desai etal from MPUH, Nadiad discuss the issues of aetiopathogenesis and management of Urolithiasis. The important point highlighted by the Karachi group is the significance of ammonium urate stones secondary to dehydration and malnutrition which can easily be prevented by providing better primary healthcare. Dr. Divyesh Desai from Hospital for Sick Children, has provided an update on the role of bladder function assessment in the management of posterior urethral valve. I feel it will be very useful for urology post graduates and the practicing urologists. The management of Wilm's tumour is a success story of oncology and today the emphasis is on curing these children with minimal morbidity. Dr. Hemant Tongaonkar, the urooncologist from TATA cancer hospital has provided a review of Wilm's tumour treatment with special emphasis on cytogenetics and various treatment modalities. Prof. E. Duardo Ruiz a famous renal transplant surgeon from Buenos Aires shares his experience of last 25 years of pediatric renal transplantation and many units who are developing the programme of pediatric transplantation would immensely benefit from this experience.

I sincerely thank all my contributors for their hard work and hope that this issue would help generate interest in this wonderful subspeciality. I take this opportunity to thank the Editorial team of IJU for having given me this opportunity.

